# Loss of embryonic vestibular ganglion neurons in a chick model of congenital vestibular disorders

**DOI:** 10.3389/fneur.2026.1721001

**Published:** 2026-02-13

**Authors:** Katherine L. Phillips, Vanshika Jain, Zoe Shaw, Nina M. Bell, Brielle Hentz, Elizabeth B. Bogin, Kathleen R. Gallagher, Anastas Popratiloff, Kenna D. Peusner

**Affiliations:** Department of Neurology and Rehabilitation Medicine, The George Washington University School of Medicine and Health Sciences, Washington, DC, United States

**Keywords:** biocytin Alexa fluor labeling, confocal imaging, congenital disorders, inner ear pathology, semicircular canals

## Abstract

People with syndromic, congenital vestibular disorders (CVDs) experience severe challenges to maintain posture and balance throughout life due to malformation of the inner ear into a sac-like structure with missing or truncated semicircular canals. How inner ear malformation affects the remaining vestibular neural circuitry is unknown, although decreased number of vestibular ganglion (VG) neurons is reported in CVD children. Our first step here was to determine whether VG neuron number is reduced before birth using our chick model for CVDs at embryonic day 13 (E13), a stage 2/3 of the way through gestation with hatching at E21. The lab implemented the ARO chick model by *A*nterior-posterior *R*otation 180° of the *O*tocyst surgically on one side in E2 chick embryos which resulted in a sac-like inner ear, the ARO chick. Estimates of VG neuron numbers, ganglion volume, and 3-D ganglion configuration were made first with classical Nissl-stained, transverse tissue sections, and second on whole-mount preparations of biocytin Alexa Fluor-labeled VG neurons in E13 normal and ARO chicks. In both preparations, VG neuron number was reduced significantly on the rotated side of ARO chicks compared to normal chicks. VG neuron cell bodies on the rotated side failed to form compact anterior and posterior ganglionic masses typical of the normal VG. Reduced ganglion cell number and abnormal ganglion topography characterized this prenatal stage, laying down a foundation for defective central vestibular neural circuitry underlying the abnormal vestibular behaviors observed postnatally.

## Introduction

The fundamental role of the vestibular system is to process signals from hair cell mechanoreceptors in the inner ear detecting head rotation, linear acceleration and changes in gravity to maintain posture, balance, and spatial orientation. Due to gene mutation or environmental insult, inner ear development may be disrupted, resulting in a congenital vestibular disorder (CVD). Children with syndromic CVD experience severe challenges to maintain posture, balance, walking, eye-hand coordination, eye tracking, reading, and language acquisition. A major hallmark of syndromic CVDs is formation of a sac-like inner ear with the three semicircular canals missing or truncated [e.g., (1)]. Normally, the semicircular canals form during the first trimester of gestation. While much is known about normal development of the inner ear and downstream vestibular circuits, little is known on how the vestibular neural circuitry develops in cases with inner ear malformation, except that the number of VG neurons is severely decreased (2–4). Known as Scarpa's ganglion, VG neurons are the first-order neurons transmitting signals from vestibular hair cells in the inner ear to vestibular nuclei neurons in the medulla oblongata. To study syndromic CVD pathology, our laboratory designed an animal model by surgically rotating the primordial inner ear or “otocyst” on one side in two-day old chick embryos (E2), resulting in formation of a sac-like inner ear phenotype observed in syndromic CVD pediatric cases (5, 6). The model is called the “ARO chick” due to *A*nterior-posterior *R*otation of the *O*tocyst 180°. In addition to anatomical malformation, ARO hatchling chicks experience balance and walking problems that mimic the vestibular clinical manifestations in CVD patients.

Our first step was to determine to what extent VG neurons survive in E13 ARO chicks, a stage 2/3 of the way through gestation when important vestibular inner ear features have formed, including the orthogonal positioning of the semicircular canals (E9–E10) and opaque-white color of the otoliths, making them excellent targets to inject biocytin and paint into inner ear structures (5, 6). In addition, E13 was selected because the principal cells of the chick tangential nucleus are readily identifiable by distinctive oval cell bodies lying in parallel with the primary vestibular fibers near the lateral surface of the medulla oblongata (7). Since the human ear achieves adult size about halfway through gestation (8), characterizing a mid-gestational stage is valuable to identify the early onset of CVD abnormalities before the full plethora of compensatory and decompensatory mechanisms interfere with achieving the mature condition. E13 is 1 week before hatching at E21, when all the vestibular sensory organs are present in the ARO sac, except for shortening of the superior crista and utricular macula in anteroposterior extent and reduced number of vestibular nuclei neurons, the principal cells of the tangential nucleus, to about 1/3 of the normal number (5). Counts of VG neurons, VG volume, and VG 3-D configuration were determined using two different approaches: (1) classical Nissl-stained, serial transverse tissue-sections, imaged by light microscopy to define VG regional topography and VG relationships to the vestibular endorgans and brainstem, and (2) more novel approach using biocytin Alexa fluor (AF)-labeled VG neuron cell bodies in whole-mount preparations combined with confocal imaging to provide higher resolution cellular images of the VG neurons, axons and other tissues within the VG mass. Since chick VG regional anatomy is not well-known, but essential to understand the relationship between the inner ear pathology and central vestibular connectivity, VG topography in E13 normal chick is presented first followed by VG organization in the E13 ARO chick.

## Materials and methods

### Animals

Cold freshly fertile White Leghorn chick embryo eggs (Gallus gallus) were purchased from the University of Connecticut Poultry Unit (Storrs, CT). Before incubation, the eggs were stored for up to 5 days at 55° F and 60% humidity in a refrigerator (Vinotemp) with little loss of viability. Cold eggs were warmed to room temperature for about 2 h before placing in a laboratory incubator (GQF incubator; Sportsman model 1502) with temperature (100°F) and humidity controls (60%), and automatic egg turner unit. All animal protocols were approved by the Institutional Animal Care and Use Committee of the George Washington University. Experiments also conformed to the International Guidelines for the Ethical Treatment of Animals.

### Otocyst rotation

Chick embryo age was determined by reference to the Hamburger and Hamilton (1951) criteria for staging chick embryos, so that the eggs were incubated for about 64–69 h to reach two days (E2; St.16) (9). After sanding down a small area of the eggshell (8–10 mm), the eggshell, chorion, and amnion were removed so that the embryo's head and right otocyst were visible *in ovo*. A fine tungsten needle was used to cut the otocyst free from the surrounding surface epithelium. The otocyst was rotated 180°, which modified the anteroposterior and dorsoventral axes of the otocyst, and then returned to the epithelial slot (5). The left otocyst was not manipulated. After surgery, the eggshell window was sealed with surgical tape (Blenderm; 3M), and the embryo was reincubated until E13, when the embryo was sacrificed for the experiment. The otocyst-rotated embryos were reincubated at 100°F, 70% humidity, without egg turning (Grumbach egg incubator) for continued development. Otocyst rotation produced a sac-like inner ear with missing or truncated semicircular canals, the ARO chick.

### Nissl preparations

E13 normal (Table 1; *n* = 4) and ARO chick embryos (*n* = 3) were decapitated and the heads immersed in Bodian's fixative for at least 2 days (10). The whole head was embedded in paraffin and sectioned in 20 μm thick, transverse serial tissue sections throughout the anteroposterior extent of the VG and stained using the Nissl method (11).

**Table 1:** VG neuron counts and volume in nissl preparations.

**Animal number**	**VG neuron count**	**VG volume (μm^3^)**
1-Normal	5,052	47.2 × 10^6^
2-Normal	5,361	43.7 × 10^6^
3-Normal	3,840	46.0 × 10^6^
4-Normal	4,521	48.3 × 10^6^
1-ARO/Intact	5,811	57.2 × 10^6^
2-ARO/Intact	5,847	75.4 × 10^6^
3-ARO/Intact	5,019	40.7 × 10^6^
1-ARO/Rotated	3,438	35.7 × 10^6^
2-ARO/Rotated	3,831	45.6 × 10^6^
3-ARO/Rotated	3,933	39.3 × 10^6^
Mean ± SEM Normal	4,694 ± 333	45.6 × 10^6^ ± 3.1 × 10^6^
Mean ± SEM Intact	5,559 ± 270	57.8 × 10^6^ ± 30.1 × 10^6^
Mean ± SEM Rotated	3,734 ± 151	40.2 × 10^6^ ± 8.7 × 10^6^

#### Light microscopy and QuPath image analysis to determine VG volume and VG neuron counts

Every third section of the VG was viewed on a Leica inverted microscope with brightfield optics and 40x objective, and LAS X application (Leica Instruments). The whole head, including the brain and inner ear, was imaged using a spiral feature (5x objective). The VG in normal chicks and those on the rotated and intact side of ARO chicks were imaged using the tile scan feature (Leica HC Pl APO, 40x/0.85 objective). Tiled images, including the entire VG, were taken on selected sections with the plane of focus chosen based on the presence of the highest number of neurons with nuclei visible in the image.

Using QuPath, whole-slide image-analysis computer software (12), VG neurons were counted on selected sections from both sides of normal chicks and the rotated and intact sides of ARO chicks (Table 1). VG neurons were identified by their large, oval cell bodies of 15–20 μm on the long axis and content of Nissl bodies. Briefly, other cells in the VG are considerably smaller and lack Nissl bodies. A counting tool was used to mark every visible ganglion cell body with its nucleus in focus in the image in normal and ARO chicks. Ganglion cell body counts were summed separately from each side on each section to give a total number of ganglion neurons in the left and right VG. The total number was then multiplied by three to account for neurons in the uncounted sections. Since only VG neuron cell bodies whose nucleus was present in the focal plane were counted, the counts do not represent actual ganglion cell numbers, but represent estimates that are valid for comparison with VGs counted using the same method in animals similarly prepared.

VG boundaries were outlined in normal and ARO chicks on the left and right sides by drawing a line along the outermost counted neuron cell bodies in the ganglionic mass using the Polygon tool in QuPath software. The scale was retained using metadata from the LAS X application. The area was multiplied by 20 μm to account for section depth when estimating the volume. Because only every third section was imaged, the section volumes were multiplied by three to include the uncounted sections. These volumes were added together to obtain the estimated total VG volume.

### *Ex vivo* biocytin-labeled preparations

Embryos (E13; normal, *n* = 4; ARO, *n* = 8) were removed from the eggshell, decapitated, and the head pinned down to SYLGARD (184 Silicone elastomer; Dow Chemical) on the bottom of a petri dish containing ice cold, oxygenated, artificial cerebrospinal fluid. The inner ear was accessed from a lateral approach using the stapes as a directional cue to quickly locate the white opaque otoliths within the vestibule. The otoliths and most of the vestibular epithelium were removed with light suction or a probe was used to break up the epithelium. Crystals of biocytin AF594 (Invitrogen) were placed on the opened peripheral ends of the VG axons for 45 min so that the tracer was internalized in the axons and transported downstream to the medulla oblongata. At the end of the procedure, the cranium was cut open on the dorsal surface, the forebrain was removed, and the remaining tissue block was immersed in 4% paraformaldehyde in 0.1 M phosphate buffered saline fixative and refrigerated. After 4–5 days, the specimen was trimmed to 3–4 mm blocks containing part of the inner ear, the entire vestibular ganglion, and the region of the medulla oblongata containing the tangential nucleus. The specimen was dehydrated in an increasing series of methanol to 100%, followed by clearing in 1:2 benzyl alcohol: benzyl benzoate (BABB), as previously described (6). The transparent tissue block containing the vestibular ganglion was placed in a depression glass slide, immersed in 1:2 BABB, and coverslipped with high precision glass (#1.5) for confocal imaging.

#### Confocal image acquisition

Confocal imaging was performed on a Leica SP8/Falcon, equipped with 3 analog photomultipliers, 2 SMD HyD detectors, and Leica HC Plan Apo lens (10x/0.4) with a working distance of 2.5 mm. Sample excitation was accomplished using 120 ps pulsed supercontinuum white laser diffracted using AOBS to 594 nm. Backscatter was further repressed by a notch filter. The emission was controlled by slits that ranged between 625 and 725 nm. To produce high quality image data, despite low numerical aperture, we employed the following approaches. All 3-D volume stacks were acquired with a pinhole of 0.3 Airy units to reduce the optical section to about 1.24 μm. The lateral pixel resolution was 2.48 μm assuming Nyquist pixel size. The images were taken in photon counting regime to ensure that very bright and very dim sample areas were registered in the lookup table without loss or saturation of the data. This required line accumulations of 6–8 line-scans to produce a dynamic range over the 400 levels of gray. Finally, combination of BABB clearing, which matches the refractive index of oil, and the above imaging strategy produced volume registrations sufficient to resolve at the level of small axons within the vestibular ganglion.

#### Imaris counts from VG confocal images

**Figure 1:**
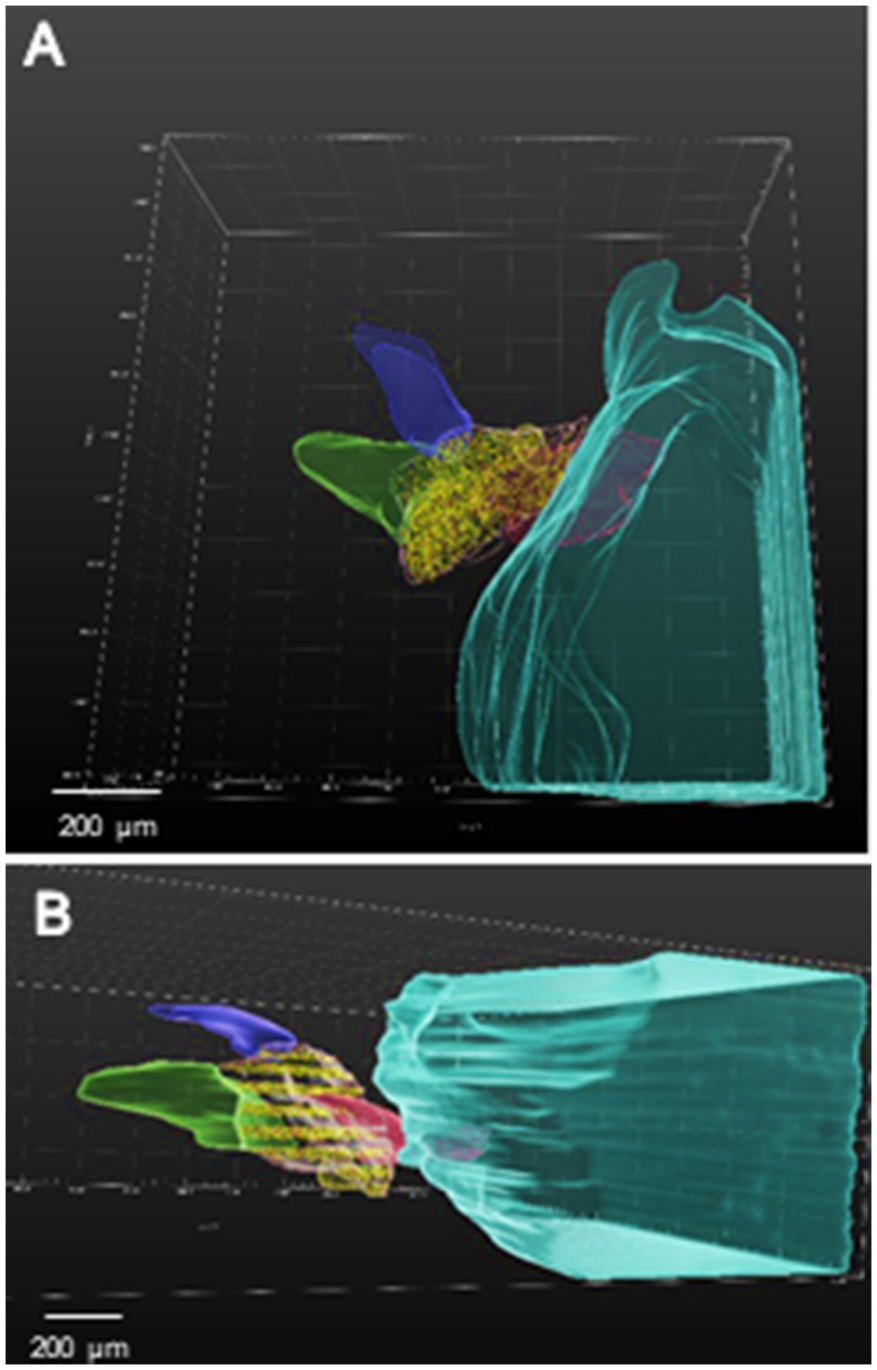
**(A, B)** 3-D Imaris confocal images of VG from E13 normal chick (Table 1; 1-Normal). **(A)** Semitransparent colors were used to define the medulla oblongata (aqua), vestibular nerve (pink) between VG and medulla oblongata, vestibular ganglion (pink line outlines VG with neurons labeled with yellow dots), lateral vestibular nerve (green) and posterior vestibular nerve (purple). The anterior vestibular nerve is not shown. **(B)** Dorsal surface of image in A has been tilted downward so that 20 μm sections containing the counted neurons appear as stripes separated by 60 μm uncounted neuron intervals.

Individual confocal images were transferred to Imaris for neuron cell body counts. The surface function was used to trace VG borders every two serial sections on the confocal image. The VG was outlined using a semi-transparent color in Mask function to visualize its 3-D location relative to other components in the image. In addition, individual vestibular nerves and the lateral surface of the brainstem were outlined on every 2 sections and assigned a specific transparent, semi-transparent, or opaque color (see Figure 1). Image contrast was adjusted to best distinguish the neuron cell bodies, axons, and lateral brain surface using the Display Adjustment tool.

The number of VG neurons in E13 normal and ARO chicks was estimated from 3-D serial confocal images using Imaris 3-D image software (version 9.8.0; Bitplane AG). Orthogonal slices with a thickness of 20 μm were collected using about 20 confocal images. VG neurons were marked with Spot tool in the orthogonal plane with every third section (60 μm spacing) counted. The VG with portions of the labyrinths, peripheral vestibular nerves and brainstem were sectioned optically primarily in the horizontal and transverse planes of section.

Starting at 0 microns in the Z-direction, image slices were analyzed for the presence of VG neurons. A VG neuron was counted if a fluorescent nucleus was observed in the neuron cell body. Counted neurons were marked by a spot (1 μm radius) placed over the nucleus using the Slicer tool. Individual neurons were marked by different colored spots using the Object ID function. Spots were viewed on the section using the Slicer tool. Serial section images of the spots were taken using the Snapshot tool. Finally, the Statistics option function counted the number of neurons labeled and identified the Z location.

#### VG neuron cell body measurements from confocal images

**Figure 2:**
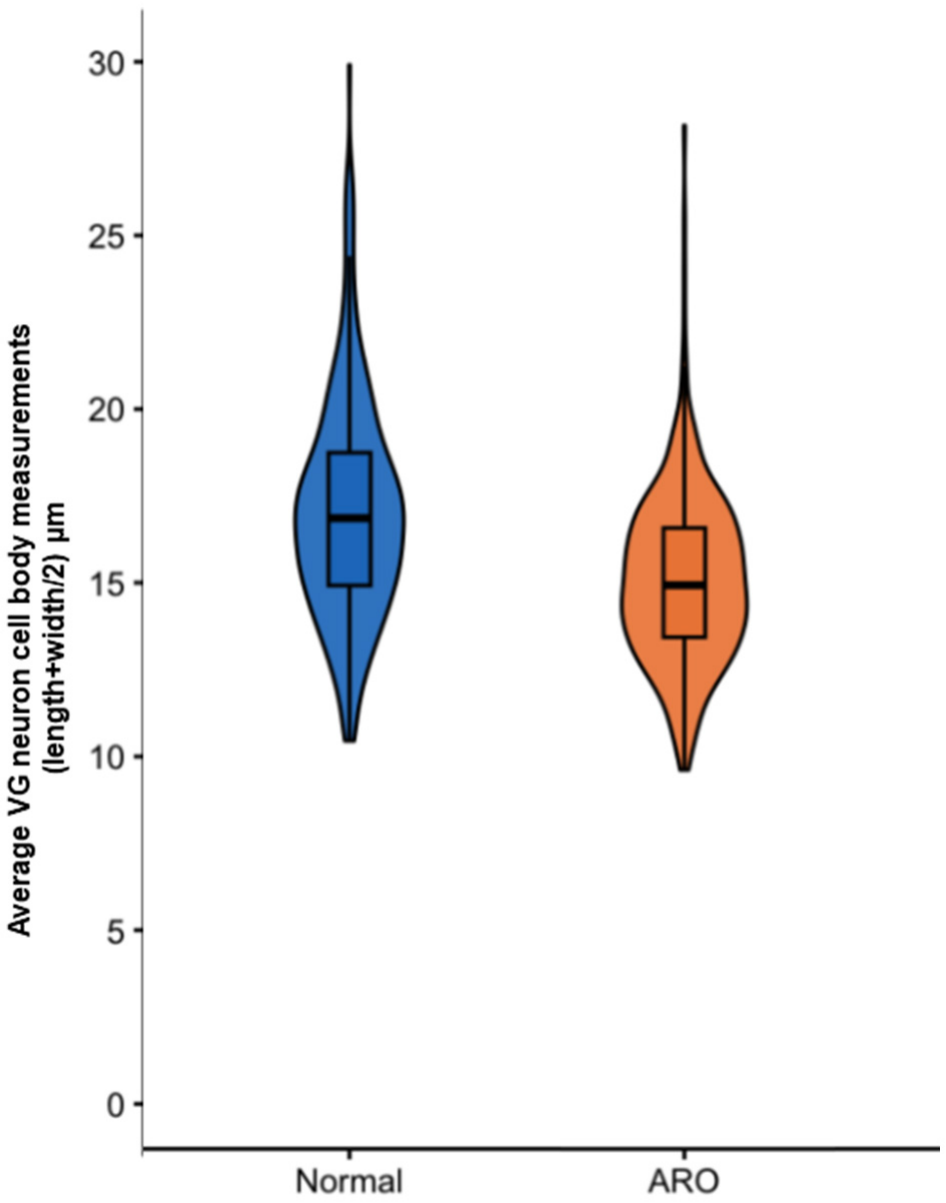
Violin plots depicting actual distribution of measurement of VG neuron cell bodies length + width/2 from normal chicks and the rotated side in ARO chicks. VG neuron cell bodies in ARO chicks were significantly smaller than those from normal chicks (Students' t-test, *p* < 0.05). Box plots inside the violins represent median (horizontal line) and upper and lower quartiles. Extended colored areas above and below the box plots show measurements above and below upper and lower quartiles.

VG neuron cell bodies were measured in biocytin-labeled preparations from normal (*n* = 2 chick; 180 neurons/chick) and ARO chicks (*n* = 2 chicks, rotated side; 180 neurons/chick) in confocal images using Imaris. The length was measured through the nucleus along the longest axis and the width was measured perpendicular to the length. No correction factors were applied to the measurements because counting errors were equivalent for all specimens prepared similarly. In Figure 2, VG neuron cell body size is plotted as length + width/2 using actual values for the measurements rather than predicted distributions in the violin plot.

### Statistical analysis

All the data are presented as mean ± SEM. Student's t-test was used to determine differences in mean values with significance set at *p* < 0.05. Data where the distribution differences were tested and non-parametric, the Mann-Whitney U-test, one-tailed, with significance set at *p* < 0.05 was used.

## Results

### VG topography and neuron morphology in normal chicks

**Figure 3:**
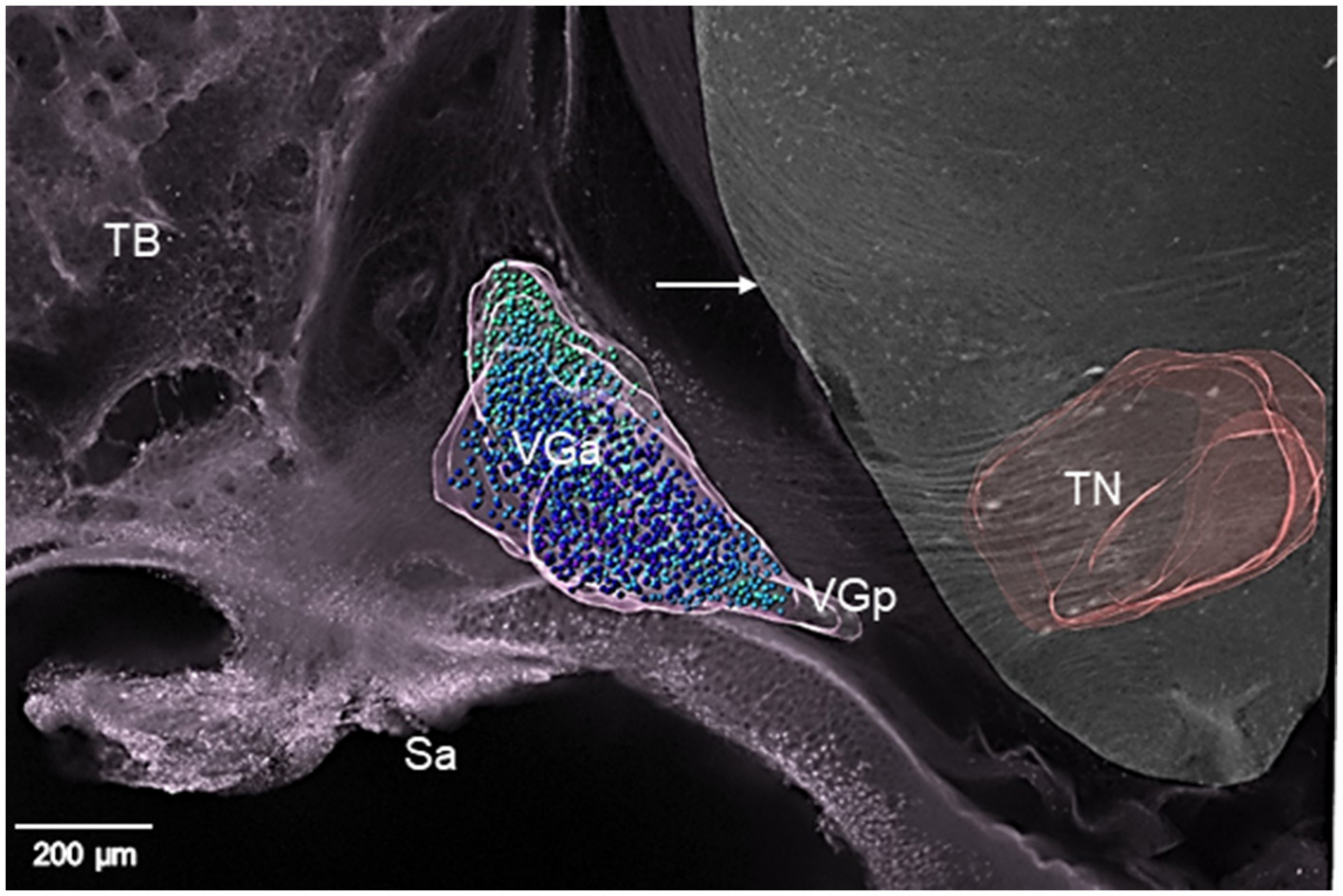
Confocal image of horizontal section depicting the topography of the anterior (VGa) and posterior vestibular ganglionic masses (VGp) from E13 normal chick (Table 1; 1-Normal). Counted VG neurons are marked by colored circles. VG and tangential nucleus (TN) boundaries are outlined by pink lines. White arrow indicates the lateral surface of medulla oblongata. Ventral is at top of the image, dorsal at bottom with lateral on left and medial on right. Sa, remnant of saccular epithelium; TB, temporal bone. Note labeled axons (white color) of VG neurons coursing through TN in parallel fiber bundles.

**Figure 4:**
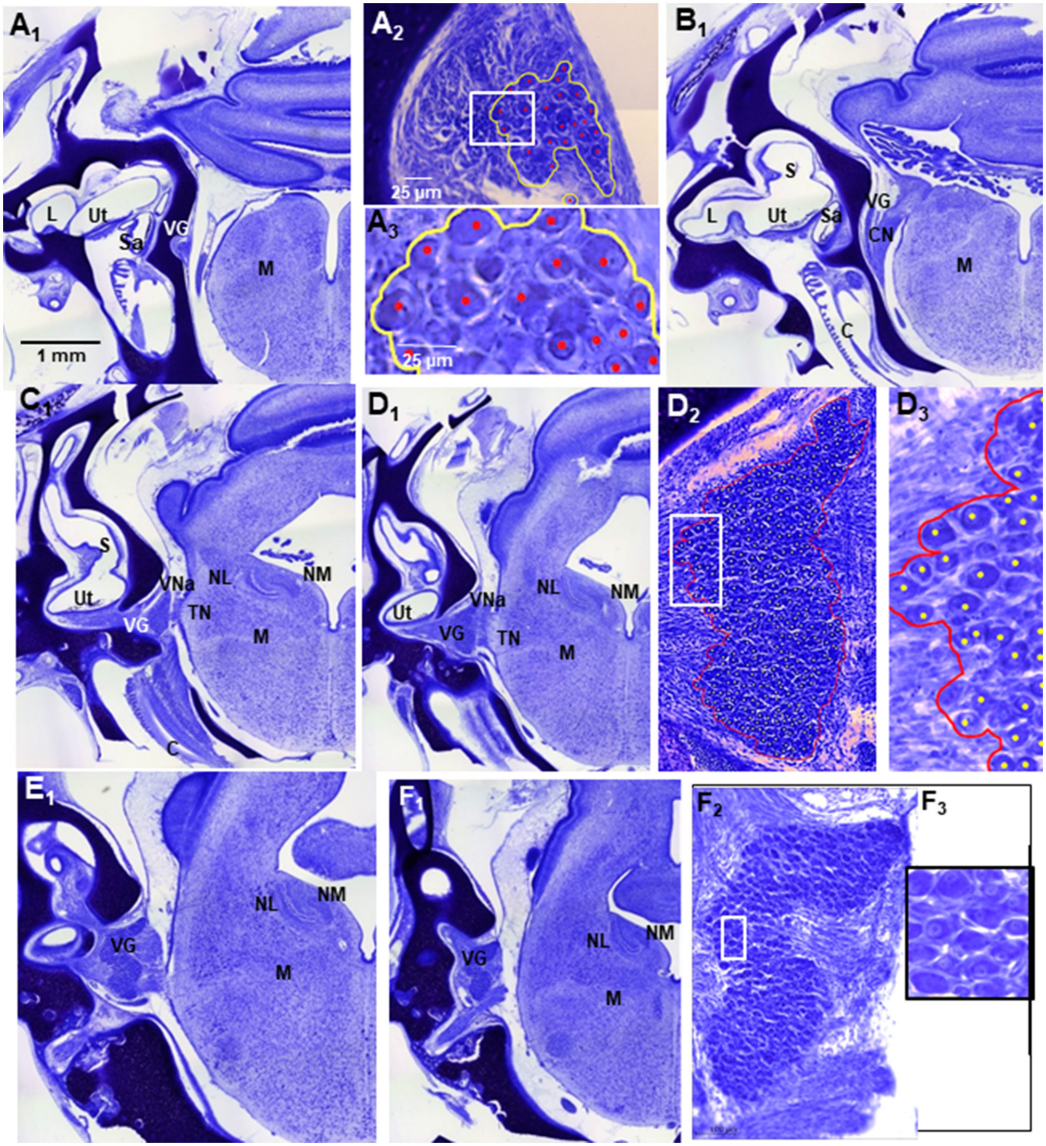
Atlas of Nissl stained, transverse tissue sections from E13 normal chick, whole-head preparation demonstrating the VG in series from posterior **(A)** to anterior levels **(F)**. In all images, dorsal is to the top; lateral is on the left. **(A**_**1**_**)** VG at far posterior level where only a few VG neuron cell bodies were found. Scale bar in **(A**_**1**_**)** refers to **A**_**1**_**–F**_**1**_. L, lateral crista ampullaris; Ut, utricle; Sa, sacculus; M, medulla oblongata. **(A**_**2**_**)** higher power image of VG with boundaries outlined by yellow line and counted neurons indicated by red dots. Scale bar in **(A**_**2**_**)** refers to **D**_**2**_ and **F**_**2**_. **(A**_**3**_**)** blocked in area in **(A**_**2**_**)** at higher magnification. Scale bar in **(A**_**3**_**)** refers to **D**_**3**_ and **F**_**3**_. S, superior semicircular canal; C, cochlea; VNp, VNa, posterior and anterior vestibular nerve branches, respectively; TN, tangential nucleus; NL, nucleus laminaris; NM, nucleus magnocellularis.

**Figure 5:**
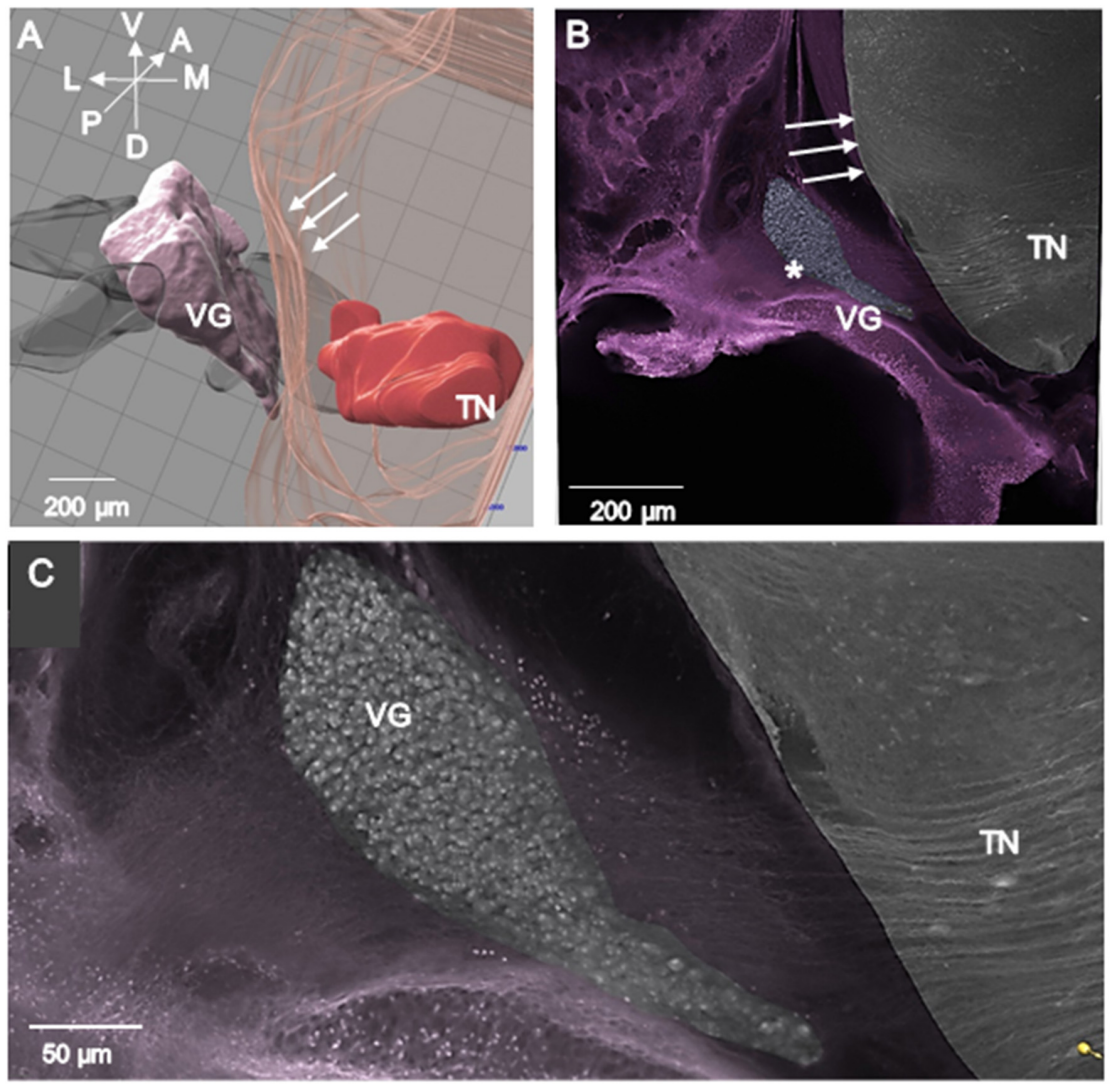
Confocal images of VG analyzed with Imaris from normal chick in horizontal sections with dorsal plane on the image surface (directional arrows in A; A-C; 1-Normal). The images are more tilted in the dorsoventral axis compared to Figure 1. **(A)** 3-D Imaris image. **(B)** Higher magnification image of VG neurons in A. **(C)** Higher magnification images of VG neurons in **(B)** to show the consolidated mass of VG neurons characteristic of E13 normal chicks.

**Figure 6:**
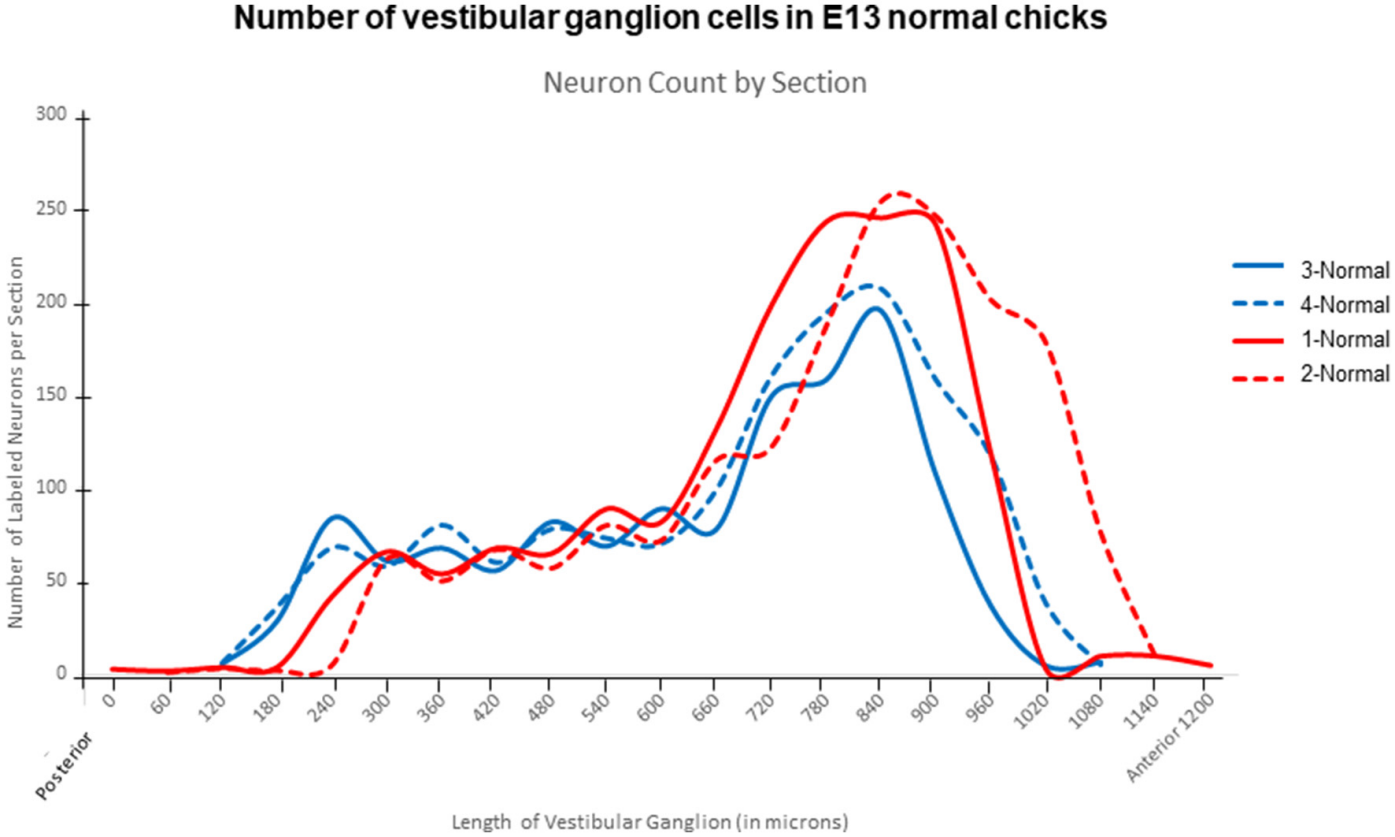
Plots of number of VG neurons in posterior-to- anterior series of tissue sections from E13 normal chicks, stained with Nissl and analyzed using Qupath program. Considerably more neurons were found in the anterior ganglionic mass than in the posterior mass.

In E13 normal chicks, the VG contained tightly packed neuron cell bodies in a large, round anterior ganglionic mass and a smaller elongated posterior ganglionic mass situated near the lateral surface of the medulla oblongata (Figures 1, 3–5) (13). In human specimens, the anterior VG mass is designated the superior part, while the posterior VG mass is called the inferior part (14). The VG is tilted in the dorsoventral plane so that the anterior ganglionic mass is ventral to the more dorsally-positioned posterior ganglionic mass (13). The posterior ganglionic mass is located lateral and adjacent to the cochlear nerve, while the anterior ganglionic mass resides within the internal auditory meatus close to the lateral brain surface, with most primary vestibular fibers entering the medulla oblongata at the level of the tangential nucleus (TN; Figures 4C_1_-D_1_). In 3-D confocal images from biocytin-labeled VG neurons in serial z-stacks, the orientation of the anterior and posterior ganglionic masses of the VG relative to each other and the lateral brain surface is distinct using semitransparent surfaces for the structures (Figures 1, 3, 5). In plots of Nissl-stained, serial sections of E13 normal chick VG in posterior-to-anterior sections (*n* = 4), the VG extends about 1200 μm (Figure 6), in contrast to 6–8 week-old postnatal chicken where the VG measures about 2500 μm (13).

**Figure 7:**
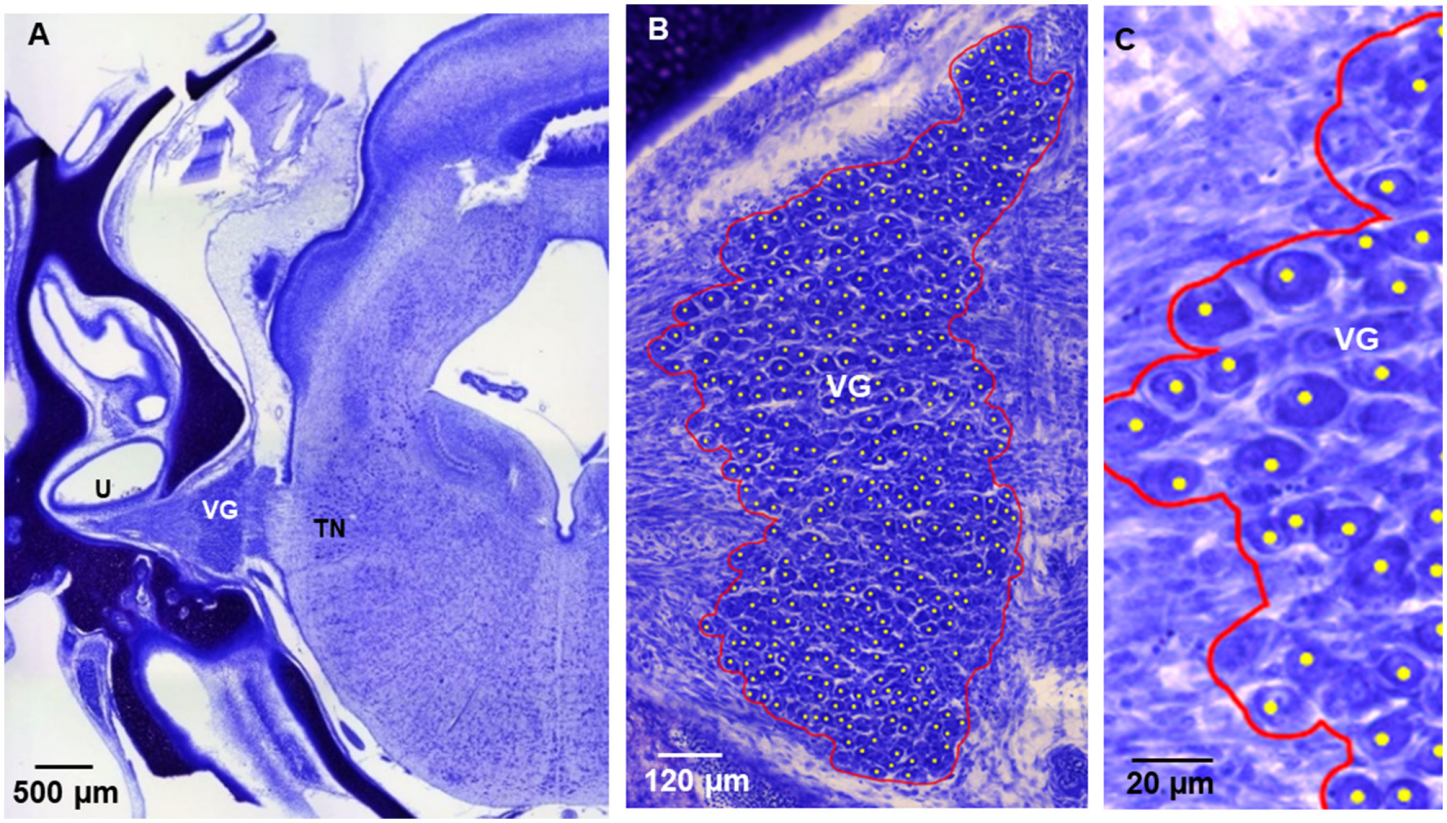
Nissl-stained, transverse section of E13 normal chick whole head, sectioned at level of central TN and utricle **(U)**. VG, vestibular ganglion; TN, tangential nucleus. **(B)** High power image of VG in **(A)**. VG boundary outlined in red was drawn to measure VG volume using QuPath. **(C)** Higher power image of VG in **(B)**. Counted VG neuron cell bodies were marked by yellow dots using QuPath. In all images, dorsal is at top; lateral is on left.

In E13 normal chicks, biocytin-labeled VG cell bodies in confocal images appeared elongated with the average size (length + width/2) of 17 ± 0.5 μm, ranging between 10 and 30 μm (Figures 2, 7). VG neurons were readily distinguished from other cells due to their selective biocytin AF labeling after transport from their cut-off axons in the vestibular epithelium where the dye was placed through axons into their cell bodies in the VG. The percentage of neurons measuring >20 μm was about 14%, while the percentage measuring < 12 μm was about 4%. These measurements are different from the results obtained from E14 chick preparations stained with Nissl, where the bipolar VG cell bodies are described as round-to-oval with 11–14 μm diameters (15). Factors, such as tissue shrinkage during dehydration and embedding media, can produce differences in neuron cell body size, so here we made all our neuron comparisons on specimens similarly-prepared.

### VG topography and neuron morphology on the rotated side in ARO chicks

**Figure 8:**
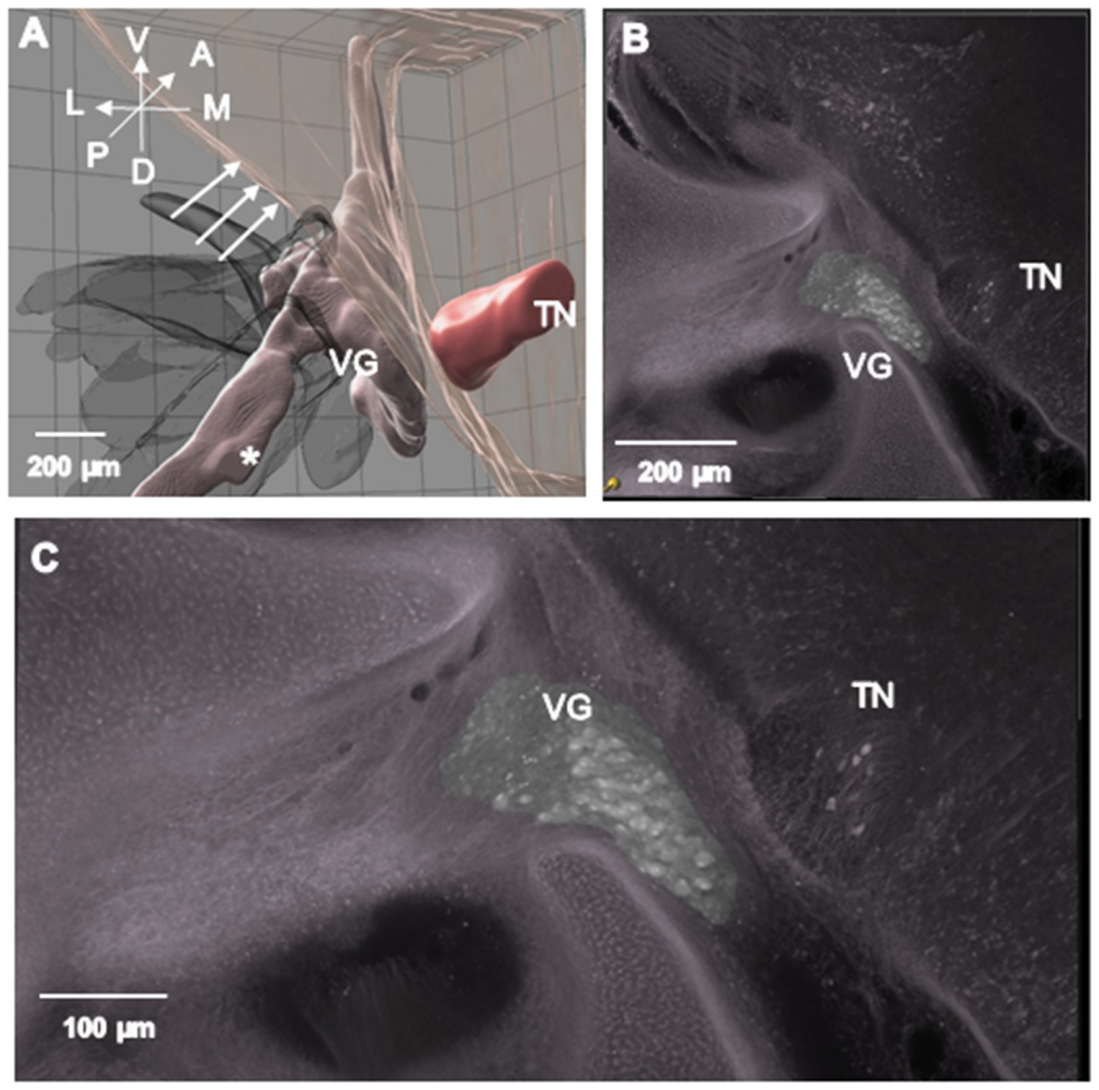
3-D Imaris image from rotated side of ARO chick. TN is labeled on its anterior surface. Series of arrows demarcate the lateral brain surface. **(B)** Higher magnification image of VG neurons in **(A)**. **(C)** Higher magnification image of VG neurons in B to show segregated VG neuron cell bodies on rotated side of ARO chick rather than a consolidated mass found in normal E13 chicks (see **Figure** 5).

**Figure 9:**
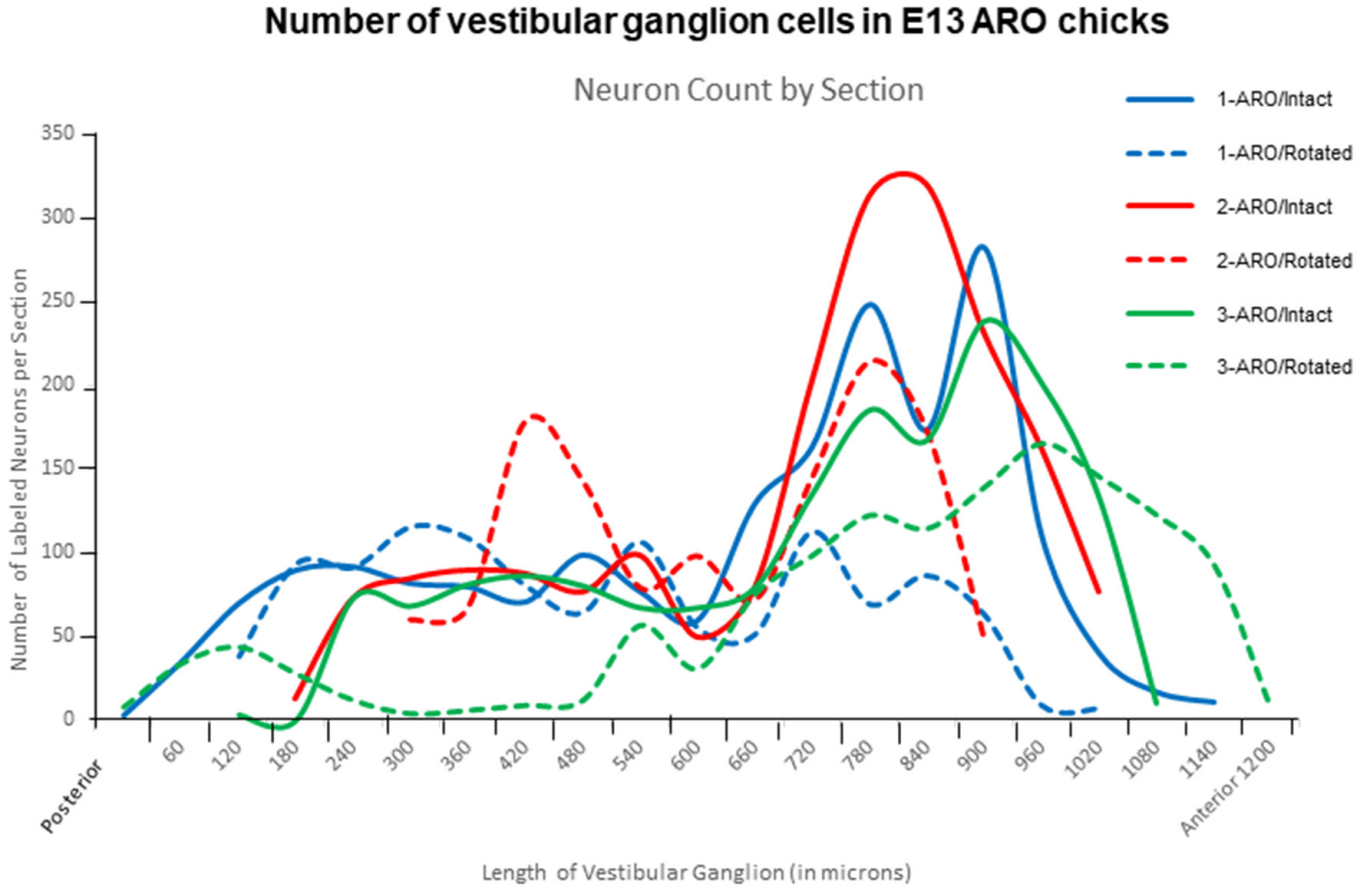
Plots of number of VG neurons in posterior-to-anterior series of tissue sections from E13 ARO chicks, stained with Nissl and analyzed using Qupath program. The number of neurons in the anterior ganglionic mass were decreased on the rotated side of ARO chicks (dashed lines) compared to the number on the intact side (solid lines) and normal chicks (Figure 5). VG neuron loss appeared greater in the anterior ganglionic mass than the posterior mass.

In comparison to E13 normal chicks (Table 1, 1-Normal), the VG was dysmorphic on the rotated side in ARO chicks (Table 2). VG neurons were not consolidated within the anterior and posterior ganglionic masses, but instead were separated by axons coursing within the ganglion (Figures 8B, C). In addition, some VG neurons were ectopic within the peripheral vestibular nerve bundles near the ganglionic masses (Figure 8^*^). In plots of VG neurons from anterior-to-posterior serial tissue sections stained for Nissl (*n* = 4), VG extent did not change appreciably on the rotated or intact sides of the ARO chicks compared to the normal chicks (*n* = 3; Figure 9). However, the number of VG neurons in the anterior ganglionic mass on the rotated side of ARO chicks was reduced compared to the number in normal chicks. A study performed on Nissl preparations from E13 ARO chicks reported that about 1/3 of the principal cell of the tangential nucleus on the rotated side are present (5). Thus, it was not surprising that the overall TN dimension in E13 ARO chicks on the rotated side were considerably smaller than that observed in normal chicks (Figure 4 vs. Figure 8; TN).

**Table 2:** VG neuron counts and volume in biocytin-AF preparations.

**Animal number**	**VG neuron count**	**VG volume (μm^3^)**
1-Normal	7,155	31.9 × 10^6^
2-Normal	7,446	83.0 × 10^6^
3-Normal	7,089	70.7 × 10^6^
4-Normal	8,781	50.5 × 10^6^
1-ARO/Intact	6,753	63.5 × 10^6^
1-ARO/Rotated	5,907	52.2 × 10^6^
2-ARO/Rotated	6,435	37.9 × 10^6^
3-ARO/Rotated	6,795	123.0 × 10^6^
4-ARO/Rotated	7,446	184.0 × 10^6^
5-ARO/Rotated	6,852	72.9 × 10^6^
6-ARO/Rotated	6,696	66.8 × 10^6^
7-ARO/Rotated	7,215	52.9 × 10^6^
8-ARO/Rotated	5,271	44.3 × 10^6^
Mean ± SEM Normal	7,617 ± 395	59.0 × 10^6^ ± 1.1 × 10^5^
Mean ± SEM ARO Rotated	6,577 ± 249	79.2 × 10^6^ ± 17.6 × 10^6^

VG neurons on the rotated side of ARO chicks measured 15 ± 0.5 μm, on average and ranged between 9 and 28 μm, so that the average cell body diameters were significantly smaller than those measured in normal E13 chicks (Students' t-test, *p* < 0.05; Figure 6). The percentage of VG neurons on the rotated side measuring >20 μm was about 2%, while the percentage of neurons measuring < 12 μm was about 10%. Altogether, these findings indicate a shift in VG neuron size with fewer large VG neurons and more small neurons on the rotated side of ARO chicks, suggesting that the larger neurons were lost and/or shrunken.

### VG neuron counts and VG volume in Nissl preparations

In normal chicks, the mean number of VG neurons was 4694 ± 333 (SEM; *n* = 4), while on the intact side of ARO chicks the average VG number was 5559 ± 270 (*n* = 3), and 3734 ± 151 (*n* = 3) on the rotated side, with significant difference between VG number on the rotated and intact sides (33% decrease; Students' t-test; *p* < 0.05), but not on the rotated side compared to counts in normal chicks (21% decrease; Students' t-test; *p* > 0.05; Table 1). In E13 normal chicks, the average VG volume was 46.3 × 10^6^ μm^3^ ± 0.9 × 10^6^, and did not change significantly on the rotated or intact side of ARO chicks (Students' t-test; *p* > 0.05; Table 1).

### VG neuron counts and VG volume in biocytin-labeled preparations

In normal chicks, the mean number of VG neurons was 7617 ± 395 (SEM; *n* = 4), while on the rotated side of ARO chicks the mean number of VG neurons was 6577 ± 249 (*n* = 8), with a significant decrease in VG neuron counts on the rotated side compared to normal chicks (13.7% decrease; Mann-Whitney U-test; *p* < 0.05; Table 2). In normal chicks, the average VG volume was 59.0 × 10^6^ μm^3^ ± 1.1 × 10^5^, with no significant difference in volume on the rotated side of ARO chicks (Mann-Whitney U-test; *p* > 0.05; Table 2).

## Discussion

### VG neuron survival in an embryonic chick model for CVDs

In the E13 ARO chick model, VG neuron number on the rotated side decreased >13% consistently compared to the contralateral side or normal, age-matched chicks using two different counting approaches. In addition, VG neuron cell bodies were disorganized on the rotated side, failing to form the consolidated masses typical of normal VG structure. Instead axon fascicle ran through the ARO VG on the rotated side among the neuron cell bodies, which also were collected into small clusters within the peripheral vestibular nerves near the main VG masses (Figure 8A^*^). In normal E13 chicks, the anterior ganglionic mass contains more neurons than the posterior mass (Figure 6), as described for juvenile chickens (13). It is interesting that the 13% VG neuron loss on the rotated side occurred primarily in the anterior ganglionic mass (Figure 9), which contains neurons contacting hair cells in the superior crista and utricular macula, those vestibular sensory organs showing decreased anteroposterior extent in E13 ARO chicks (5). VG neuron loss is supported by finding decreased number of large VG neurons measuring >20 μm, and increased number of VG neurons measuring < 12 μm on the rotated side of the ARO chicks, suggesting the neuron cell bodies may shrink during the process of degeneration.

In light of consistently reduced VG neuron number 2/3 of the way through gestation, it is notable that the remaining VG neurons nonetheless form functional neural circuitries between the ear and brain, as indicated by the presence of intact VG cell bodies that transport dye placed on their peripheral axons to their central axons in the brain. The presence of functional neural circuitries is also supported by presence of the righting reflex in ARO chicks tested shortly after hatching (5). Indeed, despite 13% loss, no change in the VG volume was found between normal and ARO chicks in both preparations, indicating a sustained degree of normality in the VG composition in ARO chicks. Our animal model sheds light on the human CVD condition by emphasizing the intact anatomical structures coexisting with abnormal peripheral vestibular features. Despite phenotype variability in CVD pediatric cases, like in ARO chicks, most CVD children are readily diagnosed early in life offering the opportunity to intervene at a young age when local neuron circuits are capable of activity-dependent plasticity. Like surgical implantation of auditory prosthesis in young children, early intervention in CVD children may be beneficial to avoid the full-blown debilitating pathology later in life (16, 17).

### Technical considerations

In this study, we applied several unique research strategies that allowed reliable reconstruction of the VG and its neurons. First, we acquired confocal images in a photon counting regime rather than using a traditional analog detection system. Although less efficient, the approach offered the opportunity to build a sufficient image dynamic range that linearly accommodated very bright and very dim sources of fluorescence without saturation. This was achieved using pulsed excitation and gated detectors capable of resolving single photons. Using longer acquisition times, the approach produced images with photon counts that sufficiently revealed very bright (site of tracer application) and very dim areas (thin axons). Second, the application of a very small pinhole was an advantage to produce robust 3-D registration despite low numerical aperture of the objective lens. Finally, the selected tracer, biocytin AF594, is transported very efficiently by VG neurons, as demonstrated in our previous study on anesthetized hatchling chicks where the tracer labeled the primary vestibular fibers centrally within 1 h (18). Accordingly, biocytin AF594 labeling used here was sufficiently strong to fully support the identification of VG neurons and their axons.

### Development of VG neurons

In the present study, otocyst rotation was performed at E2 (stages 15–17), when the otic cup has deepened and its outermost edges have fused to form the otocyst, separate from the surface ectoderm and medulla oblongata (21). The dual cellular origin of the VG has been a topic of considerable interest, resulting in an understanding that VG neurons are placodal, originating from the ventromedial wall of the otocyst (19, 20) with a smaller component derived from otic crest, a transitory structure surrounding the otic cup that disappears before St. 16–17 (21). Neural crest gives rise to non-neuronal components in the VG (19). Accordingly, the otocyst rotations performed here at E2 occur when VG neurons have already formed a separate and distinct mass from the otocyst and medulla oblongata (21), but before E3 when the peripheral vestibular nerves enter the otocyst epithelium and primary vestibular fibers enter the medulla oblongata (21, 22).

A light microscope study of the developing chick VG found a natural 24% loss of VG neurons between E8 and E14, followed by a period of stability through 14 days postnatal (15). Loss of VG neurons coincides with the time of first synapse formation by the primary vestibular fibers in the vestibular epithelium (E8–E11) (23) and brainstem with vestibular nuclei neurons (E8–E13) (24), suggesting that both peripheral and central vestibular synapses may contribute to regulating VG neuron survival and elimination in the developing embryonic vestibular system. Thus, VG neuron number was counted here at the end of the period of major neuron loss.

### Onset of semicircular canal function

In addition to developing a malformed sac-like inner ear, children with syndromic CVDs exhibit significantly decreased number of VG neurons compared to normal children (2–4). As the first-order neurons connecting the vestibular hair cells with the vestibular nuclei neurons in the brain, VG neuron survival is crucial for processing vestibular signals to maintain posture, balance, and spatial orientation. Detecting head motion depends on the fluid dynamics in the semicircular canals that convert angular head movements into fluid movements that displace the stereocilia on the apical surface of the ampullary hair cells. Endolymph fluid movement in the canals is related to key geometric features of the canals, including radius and length, so that diminished canal size and/or canal number reduces the mechanical sensitivity (25, 26). Historically, canal plugging was studied to determine the role of individual canals in generating vestibular reflex activity (27). At present, occlusion or plugging of the semicircular canals is a clinical operation performed to treat patients with symptoms due to a pathologically thin canal. Endolymph movement in the plugged canal is greatly reduced so that hair-cell stimulation is minimal, but at the same time allows for relatively normal activity in the normal canals and otoliths (28, 29). During Xenopus development, the vestibuloocular (VOR) neural circuitry is functional only after the semicircular canals acquire minimum canal diameter, despite the presence of functional activity in other components of the circuitry, including hair cells, vestibular nuclei neurons, eye motor neurons, and extraocular eye muscles (25). Taken together, the amount of fluid movement in the truncated canals on the rotated side of ARO chicks may be insufficient to bend the hair-cell stereocilia of ampullary hair cells during head movements to generate signaling, at least at low frequencies causing little fluid movement, but this remains to be tested.

### Identifying the cellular and molecular origins of CVD pathology

In syndromic CVDs, vestibular issues do not occur in isolation. However, CVD patients with other system pathologies may nonetheless achieve motor milestones. CT imaging provides information on the bony structures of the inner ear, but nervous tissues are not seen (30). In contrast, new high-resolution MRIs can image soft tissues in the peripheral and central nervous system at 25 μm spatial resolution so that some cellular abnormalities may be detected (31). Animal models offer a valuable research tool to explore human disease and accelerate the development of new treatments without compromising the welfare of human patients (31). One of the most popular and compelling animal models is the CHD7 mutant mouse that has been investigated intensively to better understand the cellular and molecular underpinnings of CHARGE syndrome [e.g., (32, 33)]. However, even with ortholog gene mutations, animal models cannot accurately reproduce the human pathology, since disease phenotypes result from complex interactions between genes and environment, and a particular phenotype results from disrupting a cascade of genes whose dosage of alleles varies [for review, see (34)]. Finally, human genetic disorders show considerable variability in the presence, type, and severity of the abnormalities so that a specific mutation may have different clinical presentations (35). The ARO chick model is constructed independent of gene mutation using microsurgical manipulation of the otocyst to produce a sac-like inner ear, as seen in CVD patients (36).

## Data Availability

The raw data supporting the conclusions of this article will be made available by the authors, without undue reservation.

## In memoriam

This paper is dedicated to the memory of June C. (Verani) Hirsch, PhD, of Paris, France, an outstanding scientist, colleague, and friend, who contributed significantly to the design and performance of this study. June passed away on April 17, 2025. Since she could not read the final manuscript, she insisted that her name be withdrawn from authorship. The study could not have been performed in its final form without her critical scientific input throughout the study, so we dedicate this paper to her memory.
